# Living with personality disorder and seeking mental health treatment: patients and family members reflect on their experiences

**DOI:** 10.1186/s40479-020-00136-4

**Published:** 2020-09-07

**Authors:** Karlen R. Barr, Mahlie Jewell, Michelle L. Townsend, Brin F. S. Grenyer

**Affiliations:** 1 School of Psychology and Illawarra Health and Medical Research Institute, University of Wollongong, Wollongong, NSW 2522 Australia; 2 Project Air Strategy Consumer and Carer Advisory Committee, Wollongong, NSW Australia

**Keywords:** Personality disorder, Lived experience, Qualitative, Consumer, Carer, Mental health services

## Abstract

**Background:**

Despite effective treatments for personality disorders being developed, consumers and carers often report negative experiences of mental health services, including challenges accessing these treatments.

**Methods:**

This qualitative study used separate focus groups to compare the unique perspectives of consumer and carers, and to investigate how to improve services for individuals with personality disorders. Reflexive thematic analysis was used to analyze the data.

**Results:**

Both consumers and carers (*N* = 15) discussed the value of providing appropriate information to consumers when they are diagnosed with personality disorder. Consumers and carers described the importance of creating a safe environment for consumers when they present to the emergency department. Both groups discussed experiencing positive and negative treatment from mental health professionals, and suggested that professionals should be trained to understand personality disorder. Limited accessibility and quality of services, and offering peer support to consumers were also described by consumers and carers. Consumers and carers also had perspectives which were unique to their group. Consumers identified the importance of psychological treatment, having a strong therapeutic relationship with a mental health professional, and the benefit of long term psychotherapy with the same professional. Broadening the scope of psychotherapies including creative, animal-assisted, and physical therapies was recommended by consumers. Carers described the importance of assessing for personality disorder and intervening early. Involvement in the assessment, diagnosis, and intervention process was important to carers. The desire to be recognized and supported by mental health professionals was discussed by carers.

**Conclusions:**

This research contributes to the concern that consumers with personality disorder and their carers experience stigma and low quality care within mental health services. In line with these findings, we recommend guidelines for health professionals who work with consumers with personality disorder.

## Background

Personality disorders are severe mental disorders characterized by disturbances in affect, identity, and relationships [1]. Approximately 7.8% of the population has a personality disorder [2], and people with personality disorders represent about 20% of emergency department and 25% of inpatient mental health admissions [3]. In mental health and primary care settings, borderline personality disorder (BPD) is the most common personality disorder [4]. Effective treatments for personality disorder exist, including dialectical behavior therapy (DBT) and psychodynamic therapies [5]. However, consumers with personality disorder often are not offered or are unable to access evidence-based therapies and thus have negative experiences when receiving mental health services [6]. In addition, carers supporting consumers with personality disorder often experience high levels of stress, grief, and mental health problems [7, 8], and they can experience difficulties accessing appropriate services for themselves and the consumers they support [9]. Considering the perspectives of consumers and carers is recommended to improve mental health services [10], and is supported by government [11, 12] and mental health professionals [13, 14].

Experiences of mental health services have been studied from the perspectives of consumers with various mental illnesses and their carers. Consumers describe the importance of mental health professionals discussing diagnoses with consumers and carers, and providing hope and information regarding diagnosis [15]. Regarding treatment planning, consumers want to be involved in their treatment decisions [16], although they are often excluded from participating in decision making [17]. Further, consumers describe not being prepared for hospital discharge, not being involved in the decision to discharge, and not receiving adequate support following discharge [18]. Carers supporting consumers with a range of mental health problems express that they have little involvement in mental health services and little access to information on mental health services [16]. Many carers believe they should have access to information about consumers, and report that confidentiality prevents them from receiving information about the consumer they support. Carers describe wanting respectful treatment for themselves and consumers, and better communication from mental health professionals, including education about specific disorders [19]. Other barriers described by carers include poor communication between services, limited service accessibility, and receiving little information about consumer treatment plans [20]. Carers also experience inconsistent assistance from mental health professionals in response to consumer mental health crises [21, 22].

Multiple studies have investigated the experiences of consumers with personality disorder regarding mental health services. Consumers with personality disorder often do not receive an explanation of their diagnosis [23], or experience stigmatizing language and insufficient evidence-based information about their diagnosis [6]. Consumers have also described negative responses from health professionals in the emergency department [24]. Other negative experiences include poor communication from professionals, and inappropriate treatment, such as not having concerns taken seriously [6]. Increasing psychological and emotional support is recommended by consumers with personality disorder [6], including being supported by health professionals who help them understand their feelings [23].

Perspectives of mental health services from carers supporting consumers with personality disorder have also been investigated. Carers often experience relationship difficulties with the consumers they support, and do not know where to find help [9]. The majority of carers want support for themselves but find carer support services are unavailable or difficult to access [9, 25]. Carers can also experience difficulty supporting consumers to find mental health professionals and consistent services to provide support to consumers with personality disorder [9]. In addition, many carers describe not receiving an explanation of the consumer’s diagnosis and not being appropriately involved in treatment decisions [25]. Carers identified wanting more information about personality disorder and how to respond to crises, and express that they are often expected to make treatment decisions without having sufficient knowledge [26].

A systematic review of 38 studies examined the perspectives of consumers and carers regarding mental health services for individuals with BPD [27]. Across the studies, consumers described receiving limited information about the assessment process and BPD diagnosis, negative responses from mental health professionals in the emergency department or inpatient setting, limited information options for therapeutic interventions, and poor communication regarding the availability of services. Studies exploring the carer perspective found that carers wanted their supporting role and their difficulties to be recognized by mental health professionals, be provided more information regarding BPD diagnosis and treatment options, and information about how to effectively respond to the consumer they support. While consumers and carers shared some consistent views, differences in opinion were observed, such as carers focusing on the lack of support they received from professionals. Comparing the consumer and carer experiences was limited because only five studies were found regarding the carer perspective, and only one study included the perspectives of consumers and carers. Therefore, more information is required regarding similarities and differences of consumer and carer views. In addition, few of the included research studies were co-produced with consumers or carers, even though this is known to lead to questions and findings closer to what people with lived experience require [28].

Problems continue to be present in personality disorder services and programs and require input from consumers and carers regarding possible improvements. It is important to increase knowledge regarding the views of consumers and carers who support someone with personality disorder, to compare views of consumers and carers regarding services for individuals with personality disorder, and to co-produce research on consumer and carer perspectives. The purpose of this study was to bring together all these needs and gaps in the literature: to explore and compare the perspectives of consumers and carers regarding personality disorder services using a co-design approach aimed to inform the development of better services.

## Methods

### Participants

Participants were recruited using a flyer advertisement that was sent to consumer and carer support and advocacy groups, and services which support individuals with personality disorder. Participants were invited to participate if they were either a consumer with a lived experience of personality disorder or a carer or family member supporting someone with a personality disorder. The views of 15 individuals were obtained, a sample large enough for data saturation within a qualitative approach. Table 1 outlines the demographic characteristics of the participants.

**Table 1:** Demographic characteristics of participants (*N* = 15)

	Consumers (*n* = 8)	Carers (*n* = 7)
Age, years: mean (s.d.), range	36.75 (17.09), 20–65	51.43 (13.61), 28–68
Gender *n* (%)		
Female	5 (62.5)	6 (85.7)
Male	2 (25.0)	1 (14.3)
Non-binary	1 (12.5)	0
Employment *n* (%)		
Work or study full time	3 (37.5)	4 (57.1)
Work or study part time	2 (25.0)	2 (28.6)
Not currently working or studying	3 (37.5)	1 (14.3)
Highest education *n* (%)		
School certificate or equivalent	1 (12.5)	1 (14.3)
Higher school certificate	2 (25.0)	0
TAFE qualification	3 (37.5)	3 (42.9)
University degree	2 (25.0)	3 (42.9)

### Procedure

Participants provided informed written consent prior to study participation, following study approval from the Institutional Review Board. Two focus group discussions occurred simultaneously; one with consumers and one with carers, as requested by the participants. Both focus groups were co-facilitated by 2 researchers with experience in personality disorders and group facilitation. The consumer focus group was co-facilitated by the consumer researcher. In addition, 1 mental health professional was present in each group to provide assistance to participants if they became distressed. Focus group questions were based on a guide that was co-designed by the authors. Some questions required participants to provide written answers or creative responses. Questions differed slightly for consumer and carer participants. Open-ended questions were followed with relevant follow-up questions as required. Questions explored the quality of care experienced at different services, including mental health services and emergency services, and how the practice of health professionals could improve. Questions included, “What have you found most helpful about the services you have been involved in?”, “Is there anything you wish clinicians and service leaders better understood about individuals living with personality disorder?” and “How can health professionals best support families and carers of individuals who have been recently diagnosed with personality disorder?” The discussions were audio recorded and transcribed. Focus group discussions occurred over a 90-min period. A $50 voucher was provided to participants as compensation for their time.

### Data analysis

The data were analyzed using reflexive thematic analysis, which conceptualizes themes as patterns based in meaning [29]. First, the transcripts were read and re-read and brief notes were made to obtain familiarization with the data. Next, participant statements were coded into nodes through the software NVivo 11. Nodes were created using an inductive orientation to gather statements with similar meanings. Themes were constructed based on the nodes, and were revised as needed to reflect the lived experience of participants. One researcher independently coded the data, which was informed by regular discussions with the research team. The consumer researcher was part of the research team throughout all phases and provided active input into the themes developed. Inter-rater reliability was obtained by all team members arriving at a consensus for the coding. In addition, an independent researcher coded a portion of data to allow us another view on agreement - with Cohen’s kappa coefficient for inter-rater reliability being κ = 0.75, which indicates a relatively high level of agreement [30].

## Results

### Consumer perspectives

In this section, the views that were gathered from the consumer focus group are presented.

#### Theme 1: challenges and successes finding a mental health professional who understands personality disorder

Consumers described how medical and psychiatric registrars often do not have the experience and knowledge base to provide treatment or information to people with personality disorders. Difficulties in finding a mental health professional who has training in and understands personality disorders were described by several participants. *“In my long hard, long lived history as a consumer, with an illness, I have been referred and searched high and low for private psychologists who would have knowledge of this particular ill – normality, this illness, and you know, there’s very few out there who are familiar enough with it.”* Consumers described various instances when mental health professionals used stigmatizing language, which had a major negative influence on their well-being. *“[After multiple stigmatizing comments] I felt so completely let down and failed by the public system. Like my life didn’t matter, like I didn’t matter.”*

Several consumers described positive experiences with mental health professionals who specialize in treating personality disorders. Consumers explained the importance of finding a mental health professional that they can connect with, who complements their specific needs. A trial and error process of finding a suitable mental health professional was described*, “They’re not always going to be – the right one is not always going to be the first one you get. There’s a lot of trial and error.”* Participants valued mental health professionals who were clear about how long they could work with them, admitted to making mistakes, kept them accountable to their goals, and persevered in contacting consumers. *“I didn’t turn up three times and she kept calling me. And she kept saying to me, ‘If you won’t come see me, let me send you to someone else.’”*

The importance of mental health professionals being specifically trained to work with people with personality disorders was discussed. *“They (mental health professionals) should all be, you know, trained to work with people who have BPD. They should understand it.”* Communicating with consumer advocates was recommended for mental health professionals to improve their understanding of personality disorders. Consumers discussed how they wanted mental health professionals to understand that people with personality disorders can recover.

#### Theme 2: the need to improve the assessment and diagnosis process

While some participants reported that they did not respond well to the diagnosis initially, others readily embraced the diagnosis. *“I liked my diagnosis. I was, like, yes. I know what it is.”* Consumers frequently described being given a diagnosis of personality disorder without any explanation or further information about symptoms, or how being provided with this information would help. One participant described receiving a diagnosis following a quick assessment, without receiving an explanation. *“I spoke to her (the psychologist) for, maybe, 60 to 90 minutes, and then she diagnosed me with borderline personality disorder… no one gave me any, sort of, information or anything. I was just stuck with this diagnosis and I knew nothing about it.”* Consumers described the potential helpfulness of receiving appropriate treatment options when a diagnosis is given, including referral to specialist clinicians. Factsheets that provide information about personality disorders, including symptoms and treatment options, were suggested to be given to consumers at diagnosis. *“I’d like a fact sheet that you could – you know. An actual, just, you – you know, this is your diagnosis, these are the symptoms you have with it, here is the available treatment options, here is what happens through with these treatment options.”* Consumers described the importance of health professionals assessing for co-occurring mental health or physical health issues.

#### Theme 3: the need to improve communication between mental health professionals to ensure continuity of care

Consumers described how improved communication between mental health professionals regarding diagnosis, treatment, and hospital discharge is needed*. “Then they referred me to the dietician who never came, and that was it, and then they just discharged me.”* Participants described disappointment when mental health professionals did not respond to recommendations made by a consumer’s private psychologist, particularly when consumers are experiencing a crisis. *“In spite of my psychologist writing a detailed letter with all of my symptoms, the fact that she’s known me for so long, and that she’s sufficiently worried about my safety at this point in time, they were still willing to try and send me back home.”* Professionals listening to the advice of a consumer’s psychologist can help consumers feel cared for and help them trust professionals and the mental health system. *“She (my psychologist) tried to visit me a couple of times in locked wards and was not allowed in, um, and – and, um, would have been really helpful if she could have just spoken to them and said, ‘Hey, here’s what works for [the person I support],’ but they wouldn’t listen to her.”* In addition, it was recommended that professionals share their resources with one another, such as fact sheets, so that consumers can receive the information they require.

The importance of continuity of care was discussed by many participants. *“He (psychiatry registrar) says to me, ‘What are you here for?’ And I say, ‘Well, did you read the notes from my last appointment here?’ He said, ‘No. Tell me all about yourself.’”* The capacity of a mental health professional to see a consumer for more than a few months may relieve a consumer from the difficulty of repeatedly sharing their past experiences. When a referral to another mental health professional occurs, it may be helpful to provide information on the consumer to assist continuity of care, if consent from consumers is provided. *“There hadn’t been any change over from the previous therapist, so we had to start all over again and tell the story for the umpteenth time.”* When referring a consumer to another service, professionals could provide some crisis skills training to help consumers while they are in between services. Following up with information that is communicated to consumers was also described as important. *“I had a lot of problems with their continuity of service, in, ‘We’ll call you tomorrow,’ and then three days later you get a call back.”*

#### Theme 4: increasing feelings of safety when consumers are experiencing a crisis

Consumers described how first responders often communicated effectively with them and helped them to feel safe and comfortable. *“I feel more safe having police and ambos come to my house than I would have an acute care worker come to my house.”* However, inappropriate verbal and physical interactions from first responders were also discussed. Consumers described how identifying with the LGBTIQA+ community can result in negative or poor treatment from some first responders.

Some consumers described how acute care units and emergency departments did not provide a safe environment. Consumers described receiving negative judgments from mental health professionals during crises, including being ignored, shamed, denied services or being told that they are *“not trying hard enough.”* Simple changes to service environments such as allowing curtains to be drawn or receiving positive communication from mental health professionals were described to increase comfort. *“They allow you to have the curtains on, so you can calm yourself down.”*

Limitations of inpatient wards and emergency departments were described, such as being locked up and alone. Therefore, alternative safe places were suggested for consumers to go to when experiencing distress, such as cafes, respite homes, or rehabilitation centres. *“I can’t be alone because I’m not safe enough to be alone, but I don’t need the acute care centres. I just don’t even need to be talking to someone, but I just need to not be alone.”*

#### Theme 5: providing expanded treatment options and increasing service accessibility

Consumers described various ways that treatments and services could be improved. Some consumers discussed the power of art therapy and creative therapies, animal-assisted therapy, nature therapy, and physical therapy. *“I found a sexual assault nurse who actually got balloons and filled them with, um, like, paint, and just gave me, like, darts, basketball shooters, the room was just splattered everywhere. It was so colourful that it was a distraction… I find sometimes just having a psychologist isn’t good enough, you need that art therapy; you need the physical therapy.”* Several consumers described how peer support could aid them, including providing support groups and safe places where people with lived experience can connect. Consumers also described the helpfulness of 24-h phone lines. One consumer described the usefulness of e-therapy. *“He (my psychologist) was prepared to do some sessions remotely by video-conference. You know, so, we were just about to go into the UK at the time, and then it – I didn’t have to break my therapy.”* One consumer discussed the benefit of support being provided to carers. *“The support group that my mum has been going to… before she’d often just get upset or angry or - whereas now she just seems to be a lot better at knowing what to do without making it worse, kind of thing. So, it’s good.”*

Consumers discussed the limited availability of mental health services for personality disorders. Some barriers to accessing mental health services included homelessness, location, and finances*. “I wish clinicians understood how cost-prohibitive consistent treatment is for low-income patients.”* Non-government organizations were acknowledged by some participants as providing better care compared to government organizations.

### Carer perspectives

In this section, five themes from discussions in the carer focus group are presented.

#### Theme 1: the importance of carer involvement in early assessment and intervention

Carers described how they wanted to be involved during assessment, diagnosis and intervention. Receiving a diagnosis for the person they support was described as taking a lengthy amount of time. “*My biggest issue was getting the diagnosis. Yeah. That took 10 years. Yeah. And the hardest part was that how quick they seem to have – have wanted to keep sending her home.”* Frustration was expressed by carers about how mental health professionals often mislabelled a consumer’s difficulties as anxiety, depression, or ‘normal’ behaviours, before later giving a diagnosis of personality disorder. Carers described working hard to find a mental health professional who would provide an assessment or diagnosis for the person they support, particularly during adolescence. *“It took yeah, begging and pleading and we are not taking her home until we spoke to a psychiatrist, to tell them our side, and then we got a diagnosis.”* After diagnosis, carers emphasized the importance of mental health professionals explaining a personality disorder diagnosis to consumers and carers. The importance of early diagnosis and assessment was highlighted by many carers, such as when a person first experiences a crisis. “*They hit their absolute lowest before there’s a click or a diagnosis into what’s going on, in comparison to trying to seek help for many years, when you can already see many traits*.”

Carers discussed the importance of communicating their perspective of the person they support to inform decisions made by mental health professionals, such as diagnosis. Involving carers as soon as possible was recommended, such as during the consumer’s first crisis. Several participants suggested involving carers in treatment helps them to understand what the person they support is learning and experiencing. Confidentiality was described as a barrier to carers being involved in assessment and treatment. *“There is no communication, because of this confidentiality. And I think that could be the worst enemy, basically, standing in the way of the family therapy.”*

#### Theme 2: improving responses and follow-up when consumers present in crisis

Mixed feedback was received from carers in relation to the responses from police and ambulance responders. Some negative interactions were described, including physical force by police rather than a dialogue approach. *“In one case, the police came and basically… he was thrown on to the floor, you know, with policemen with the guns. It was so traumatic, instead of first having a dialogue approach.”* However, many carers described compassionate treatment from police and ambulance responders towards consumers and carers, which was sometimes experienced as comparably better than treatment provided by other mental health professionals during crises. *“The first responders are much more caring for carers, family members and explaining what they’re doing, and in their compassionate treatment.”*

Carers discussed how consumers can experience difficulties at the emergency department when there are physical health assessments and long wait times for mental health problems. Several carers discussed how separating mental health problems and physical health problems in the emergency department may result in better care. Providing a safe place within the emergency department *“that people can go to in a crisis to calm down and self-soothe”* was also recommended.

Carers described how consumers were often sent home from the emergency department without appropriate support. *“We went to emergency and were sent home with nothing in our first instant… [the person I support] was just sent home to me, with no explanation of anything*.” Following discharge from emergency departments or inpatient services, carers recommended that mental health professionals inform consumers and carers about the treatment that was provided and treatment options for the future. Carers proposed that communication between mental health professionals and carers about a consumer’s hospital discharge can help protect the safety of consumers and others. Several carers described not receiving information from mental health professionals unless it was requested by the carer. *“Even when [the person I support] was sent home from hospital two times, she was never sent home with anything… Not unless you ask for it.”*

#### Theme 3: increasing mental health professionals’ understanding of personality disorders and improving communication

Carers described the harms of mental health professionals using inappropriate and stigmatizing language when communicating with consumers and carers. The use of recovery-oriented, strengths-based language was desired by carers, such as expressing an understanding of the difficult experiences faced by carers and consumers. Mental health professionals who provided explanations about mental health problems which can be understood by consumers and carers were valued. *“I think if they actually remember that this is the first time someone’s hearing it, they actually may be forthcoming with more information.”*

Many carers discussed improving training and awareness of personality disorders for health professionals. *“If the training is proper – with the GPs, with the doctors, psychiatrists, psychologists, nurses, we have the system right. It’s a matter of just the right education.”* Carers described how mental health professionals need to be aware of support that is available and to explain treatment options. Carers also wanted guidance from mental health professionals on how they can best support consumers. *“We (carers) need to know what we can do to help them. We want to understand how they feel and why they act/behave the way they do. Please help us to ensure they get the best care and the treatment they need to recover.”*

Carers discussed the value of mental health professionals communicating with one another, including providing referral information. When mental health professionals liaise, it can provide a more holistic picture of a person’s difficulties, including physical and psychological symptoms. *“[The person I support] has a lot of physical symptoms that I think are a result of her mental state. But I’m not sure. So, they sent her off for all these tests… but there’s no – no one’s like, pulling it all together. The GPs should be, but they don’t.”*

#### Theme 4: improving accessibility and quality of services for consumers

Several carers described limited availability and quality of services within the mental health system, including the public and private healthcare system. *“She’s had stays in private hospitals as well. And to be honest, not a lot better. I mean, it’s much nicer place. But I don’t know that the level of care is much better, really, considering how much you pay for it.”* The small amount of psychological sessions provided by the public healthcare system was described by carers as insufficient. The proximity of services was also described negatively, including consumers having long commutes to receive treatment. Long wait times to receive treatment were also discussed. *“She was on four waitlists in the city at private clinics. One down here in this region. Couldn’t get her in. Christmas Eve, they rang and said, ‘Oh, we’ve got a bed in the city.’ So October, November, December, she was on 24-hour watch. Because I couldn’t get her in anywhere.”*

Carers also recommended personalizing therapy for specific consumers, including offering support in nature. Several carers described the helpfulness of DBT. *“She (the person I support) ended up being put through a DBT group… that has by far been one of the best things for our entire family.”* Carers described how offering employment assistance and peer support groups for consumers may be beneficial.

#### Theme 5: improving support for carers

Carers described feeling overwhelmed and stressed by caring for a person with personality disorder and suggested carer respite as a valuable form of support. One carer described feeling hopeless after multiple attempts to find a treatment that would work for the person they support. Financial and work difficulties due to time commitments supporting someone with personality disorder were also described. *“The Government needs to know is the financial strain on families… with needing weekly psychologist, regular psychiatrist, not being able to get to work, because you get called home all the time.”*

Carers discussed the importance of mental health professionals understanding the difficulties experienced by carers. Carers described receiving little support for themselves from mental health professionals. Mental health professionals asking a carer ‘how are you?’ was described as a positive first step. *“I had one registered nurse, who was special… who actually asked me how I was. And that was probably year six of the journey. And until then, not a soul had ever asked me how I was.”* Other options for providing support to carers were discussed, including a 24-h phone line, peer support groups, counselling for carers, and promoting self-care. Providing educational resources to carers was recommended, such as having brochures in hospital waiting rooms, offering educational groups, and providing links to online information. Several carers recommended increasing public awareness and understanding of personality disorders through education, which may help others in the general community understand the experiences of consumers and carers.

A comparison of the consumer and carer themes can be found in Table 2. Both consumers and carers described disturbing stigma and prejudice, but also receiving some exemplar care from some professionals. Broadening support options for both consumers and carers was a priority.

**Table 2:** Similarities and differences between consumer and carer themes

Theme subject	Consumer themes	Carer themes	Similarities	Differences
Assessment, diagnosis, and intervention.	The need to improve the assessment and diagnosis process.	The importance of carer involvement in early assessment and intervention.	• Consumers and carers described the importance of providing information to consumers when a diagnosis is given.	• Consumers described the potential helpfulness of receiving appropriate treatment options and referral information at diagnosis. • Carers described the importance of carer involvement in assessment, diagnosis, and intervention. • Carers described the importance of early assessment and intervention.
Mental health professional’s understanding of personality disorder, communication between professionals, and continuity of care.	The need to improve communication between mental health professionals to ensure continuity of care. Challenges and successes finding a mental health professional who understands personality disorder.	Increasing mental health professionals’ understanding of personality disorders and improving communication.	• Consumers and carers described the importance of professionals communicating with one another to better understand the consumer and provide continuity of care. • Consumers and carers suggested improving training for mental health professionals to increase understanding of personality disorder. • Consumers and carers described receiving stigmatizing responses from mental health professionals.	• Consumers described the value of finding a mental health professional that they can connect with. • Consumers described the benefit of having a long term relationship with a mental health professional. • Consumers described the importance of other professionals listening to their private psychologist’s advice.
Services and responses for consumers experiencing a crisis.	Increasing feelings of safety when consumers are experiencing a crisis.	Improving responses and follow-up when consumers present in crisis.	• Consumers and carers described receiving compassionate treatment and some inappropriate treatment from first responders. • Consumers and carers described how the emergency department was often not a safe environment for consumers.	• Consumers described the potential benefit of providing alternative safe places that they could access when feeling distressed. • Carers described how consumers and carers are not provided sufficient information or support following presentation to acute care services.
Expanded treatment options, and service accessibility.	Providing expanded treatment options and increasing service accessibility.	Improving accessibility and quality of services for consumers. Improving support for carers.	• Consumers and carers described insufficient availability and quality of services for consumers. • Consumers and carers described how offering peer support to consumers may be beneficial.	• Consumers described the potential benefit of providing options such as creative or animal-assisted therapy. • Carers described difficult experiences supporting a consumer with personality disorder and suggested increased support for carers.

## Discussion

This study explored and compared experiences of personality disorder services from the perspectives of consumers and carers. Consumers and carers described a number of negative and positive experiences with mental health services and provided recommendations on how services could improve.

Both consumers and carers discussed the importance of receiving appropriate information when a person is diagnosed with personality disorder, which is consistent with previous research [6, 23, 31, 32]. Taking a collaborative stance in working with consumers during the assessment and diagnosis process was identified as a way to reduce stigmatization and empower consumers to engage in treatment [33]. Safety when in crisis was a major concern, both interpersonally (e.g. through promoting compassionate communication) and physically (e.g. avoiding rough handling by authorities, having safe rooms within emergency settings). Consumers also described creating safe places separate from the emergency department, such as voluntary residential or drop-in programs. Previous research indicates that residential programs may be a beneficial alternative to the emergency department for consumers with BPD [34]. Investigating the carer perspective of alternative safe places and respite options could also be important.

Both positive and negative experiences with mental health professionals and first responders were described by consumers and carers. The literature suggests that stigmatization and discrimination of personality disorder in mental health services continues to be prominent [35], although professional attitudes toward personality disorder have improved over time [36]. During crises, consumers and carers expressed receiving better treatment from first responders, compared to mental health professionals, which may mean that mental health professionals have more stigma of personality disorder compared to first responders [37]. Both groups suggested improving mental health professionals’ knowledge and understanding regarding personality disorders. Research has shown that training can improve mental health professionals’ understanding and attitudes [38, 39]. Increasing the accessibility and awareness of training may be required. Providing training to first responders and increasing public awareness of personality disorder may also help reduce stigma and discrimination [35].

Consumers and carers described the potential benefit of offering peer support to consumers. Peer support can help consumers with various mental health problems by providing shared experiences which offer validation and hope [40, 41]. Increasing the number of peer workers and peer support groups for consumers with personality disorder may be valuable. In addition, both carers and consumers described difficulty accessing personality disorder services. Increasing availability of services and making services more affordable may benefit consumers and carers.

From the consumer perspective, importance was given to the therapeutic relationship with mental health professionals, including finding a clinician they can connect with who specializes in personality disorder. This finding is unsurprising given that a strong therapeutic alliance can facilitate recovery [42]. Consumers described how mental health professionals, such as medical registrars, often did not have sufficient knowledge and experience to support them, and they requested people with experience who had specialized knowledge of personality disorders. Ensuring registrars who work with consumers with personality disorder have appropriate knowledge prior to in-person interactions and are supported by a specialist mental health professional may be helpful. Regarding referrals, health professionals should increase their awareness of personality disorder treatments available in their area, and offer consumers a range of possible mental health professionals that can support them.

Consumers also discussed the importance of continuity of care, including being able to work long term with a mental health professional. Therefore, it is important for mental health professionals to clarify how long they can work with consumers and to provide appropriate support when a consumer is transitioning from one professional to another. In addition, mental health professionals should communicate with one another to ensure they have all necessary information to support a consumer with personality disorder. Collaboration amongst mental health professionals involved in a consumer’s treatment is associated with improved consumer outcomes [43]. Further, consumers described the importance of private psychologists being able to communicate with other professionals involved in their care during a crisis. With a consumer’s consent, emergency department and inpatient services should collaborate with a consumer’s primary mental health professional, such as a private psychologist.

Expanded therapy options, such as art and animal-assisted therapy, were also recommended by consumers. Previous research has shown that art therapy can help increase well-being and decrease symptoms in consumers with a personality disorder [44]. Increasing accessibility and affordability of creative therapies and other approaches is recommended to improve referrals and options for consumers.

For carers, importance was placed on early assessment and intervention, which is supported by evidence and treatment guidelines [10, 45]. However, consumers did not discuss early intervention, although they have previously described delays in receiving a diagnosis [42]. Carers also focussed on being involved in the assessment and treatment of the consumers they support, although consumers did not mention this. While carer involvement in assessment and intervention is important to carers and may help them support consumers [46], the perspective of consumers should be considered because not all consumers endorse family or friend involvement in their care [47, 48]. In addition, carers focussed on improving support for carers, including having mental health professionals checking in on carers. However, consumers can have negative experiences of mental health professionals providing support to their carers [47]. Therefore, professionals may need to find a balance when providing support to consumers and carers. For example, a professional might provide carers with referral information to a psychological education or carer peer support group after consulting with the consumer and clearly explaining the reason for providing support to carers. Alternatively, carers may seek their own supports through mental health professionals and support groups. Table 3 provides a summary guideline of recommendations for health professionals arising from this research.

**Table 3:** Guidelines for health professionals who support consumers with personality disorder

Attend training to enhance understanding of personality disorder.	
Ask consumers if anything can be done to increase their feelings of safety when they present to the emergency department.	
Consider creating safe places for consumers within and outside the emergency department.	
When consumers are discharged from the hospital, provide carers with information where appropriate, including facts regarding increased suicide risk following discharge and 24/7 crisis services. With consumer consent, inform carers of the consumer’s safety plan and outpatient treatment plans.	
Provide information to consumers when giving a diagnosis of personality disorder, and provide information to carers if appropriate.	
Increase accessibility of services for consumers with personality disorder.	
Be aware of local services and treatments for consumers with personality disorder, and provide appropriate referrals and treatment options to consumers.	
Inform consumers how long you can work with a consumer.	
With consumer consent, communicate with other health professionals who are supporting the consumer to ensure you have a holistic understanding of the consumer.	
Consider offering or referring consumers to other treatment approaches, such as art therapy or peer support.	
Where appropriate and with consumer consent, encourage carer involvement in assessment and intervention.	
Offer carers information regarding carer support services, such as support groups.	

### Limitations and future research

Although data saturation occurred in the analysis of qualitative interviews, the small sample size used in the study may be a limitation as other views may not have been represented [49]. We did not investigate further the treatment history, specific diagnoses of the consumers, amount of carer engagement with services, or cultural background of participants, meaning it was difficult to estimate to what extent our sample were representative of the broader consumer and carer population. Statements spontaneously reported by consumers and carers did reflect in detail findings from previous studies supporting that our sample was comparative to others in the literature. Further, the sample was predominately female, and the perspectives of male consumers and carers were limited, and it would be important to increase their participation in future research. Consumers and carers were not always asked the same questions, making it difficult to compare their experiences in some topic areas. The groups were ran as semi-structured focus groups and the facilitators followed a guide, but were all responsive to the participants in the focus group and what they wanted to focus on. For example, consumers were not asked about carer involvement in assessment and treatment. Despite the limitations, the findings provide important information to improve services for individuals living with personality disorder and their carers. Future research could explore safe environments for consumers experiencing crisis, and expanded treatment options for personality disorders, including art therapy and peer support. In addition, there is a need to broaden our understanding of the variety and nature of consumer views of having carers involved in their assessment and treatment.

## Conclusions

The current study explored and compared mental health service experiences from the perspectives of consumers with personality disorder and carers. The findings add to the ongoing concern about the stigma, prejudice and poor provision of services for people with personality disorder, despite some examples of high quality work being delivered. In addition, the findings highlight similarities and differences in consumer and carer perspectives. Based on the findings, a number of guidelines are provided to inform the practice of health professionals who support consumers with personality disorder.

## Data Availability

Data from the current study will not be made available, as participants did not consent for their transcripts to be publicly released. Extracts of participant responses have been made available within the manuscript.

## Abbreviations

BPD: Borderline personality disorder
DBT: Dialectical behavior therapy
